# Private Anatomical School, at Auburn, New-York*Introductory Address and Catalogue of Students attending the Annual Course of Lectures on Anatomy and Surgery, delivered by F. H. Hamilton, M. D. Auburn, 1837.

**Published:** 1837

**Authors:** 


### Art. IV.—Private Anatomical School at Auburn, New York.*

Every teacher of the theory and practice of medicine, must have had occasion to lament the defective state of anatomical

---

* Introductory Address and Catalogue of Students attending the Annual Course of Lectures on Anatomy and Surgery, delivered by F. H. Hamilton, M. D. Auburn, 1837.

knowlege in which pupils are sent up to him to be instructed in pathology; and all will feel gratified, at every additional effort to impart a better understanding of anatomy and physiology to our medical novitiates. The private school of Dr. Hamilton, in this beautiful village of New York, (we recollect it as beautiful,) is of this kind. His class of last autumn numbered 43, the names and residences of the whole of whom are printed. Such an establishment must do much good to the students of the surrounding country, and ought to be encouraged by every physician who lives near enough for his students to visit Auburn. We regret, however, to read in the Address, that the lecturer is under the necessity of limiting his demonstrations to colored engravings, preparations, and dissections in comparative anatomy. Much may, it is true, be done by these instrumentalities; but the dissection of the human body would, of course, be incomparably better;—and is it possible, that the people of the Great State are so delivered over to prejudice and superstition, as to interfere with the only true and successful method, of studying one of the most important of all the sciences? Why do not the governors of the Auburn Penitentiary, make it subservient to the cause of medical science? Why do not the citizens unite in facilitating, for anatomical purposes, the acquisition of the bodies of those who die without friends?

The Address of Dr. Hamilton shows him to be a man of sense; and, what is not less important in a public teacher, of devotion to his object. The following page contains views which do him credit:—

"The position we hold, and which, if we stood alone, we should steadily maintain, is, that pathological anatomy is the only basis of correct theory; the chart and compass of successful practice; and that as well might the mariner dare the dangers of the ocean without a helm, as the physician enter his profession without an ample store of pathological knowledge. He may, indeed, with caution, lurk along the shore, when the winds are low and the sky unclouded, and save the few whom the late tempest has thrown within his reach, but can never venture forth with boldness to rescue those who are yet rudely rocked by the tempestuous winds, when the storm and the night are dark. We do not allow, in this matter, every man to judge; physicians only, and of these a limited number, are competent; because physicians only, and of these *not many*, are conversant with anatomy and pathology; and we hold no man competent to judge or censure an opinion with which he has not, neither can have, any acquaintance: and we could wish that with such men, the reproof once given to a superstitious bigot, indulging in vague invective against the doctrines of a certain unpopular author, might produce a similar effect; on being asked whether he had read the work he so much derided, he was suddenly struck dumb, and became fired with silent resentment. But such is not often the happy effect. Men ignorant of that with which they are conscious they should be acquainted, generally assume an appearance of knowledge and vast show of argument, which to those unacquainted may have such a seeming. Hence the argument, plausible enough, but unfounded, that the science of anatomy cannot guide us to the measure or kind of medicine we should administer; the mode or time of its exhibition; which argument we meet with the broad assertion of its falsehood, and are prepared to support the assertion by a series of demonstrations in the course, to which this lecture is introductory. We shall also not fail to show by the same demonstrations, that the practice of the pathologist cannot but be more successful than that of the mere experimentalist. Who will be the most successful chemist, he who merely throws together the ingredients, and observes the fermentation and decomposition which ensues, or he who examines the elements and ascertains by actual analysis what are the primary constituents of the substance acted upon, and infers from their form and character the exact kind and amount of the chemical required to act most forcibly and certainly?

If any are silly enough to contend that pathology and physiology alone are necessary to the physician, and that these may be acquired without a previous intimate acquaintance with anatomy, we are only amused at their ignorance. How can a man learn the function of an organ and the manner in which that function is performed, unless he first learn its form and structure? or how can a man be taught what appearances are diseased, unless he first be taught what appearances are normal and healthy?"

Writing at so great a distance from the theatre of Dr. Hamilton's labors, we cannot hope to benefit *him*, but the promulgation of this notice in the WEST, may give effect to his example in a region where it may be followed with fewer obstacles, we believe, than he is encountering; and should this opinion prove to be well-founded, we shall not have written in vain.

In reading the proof sheet of this article, the thought occurs to us to notify our country friends that Professor McDowell is engaged in making plaster casts of the organs of the abdomen, chest, and brain; and would be able to furnish them to any one who might apply. They would be particularly valuable in academies, male and female, where an outline of anatomy is taught. D.

